# Undergraduate Medical Education Reform in Viet Nam for a Primary Health Care Workforce

**DOI:** 10.5334/aogh.3755

**Published:** 2022-11-09

**Authors:** David B. Duong, Tuan A. Nguyen, Kristen Goodell, Nora Y. Osman, Tam M. Nguyen, Van-Anh T. Pham, Loan T. Vu, Hong-Anh T. Vu, Lisa A. Cosimi, Todd Pollack, Barbara Gottlieb

**Affiliations:** 1 Program in Global Primary Care and Social Change, Center for Primary Care, Department of Global Health and Social Medicine, Harvard Medical School, Boston, Massachusetts, US; 2 Pediatrics Department, Faculty of Medicine, University of Medicine and Pharmacy at Ho Chi Minh City, Ho Chi Minh City, Vietnam; 3 Boston University School of Medicine, Boston, Massachusetts, US; 4 Harvard Medical School, Director of Student Medical Education, Department of Medicine, Brigham and Women’s Hospital, Boston, Massachusetts, US; 5 Family Medicine Department, Hue University of Medicine and Pharmacy, Hue City, Vietnam; 6 Practice of Medicine Program, Hai Phong University of Medicine and Pharmacy, Hai Phong City, Vietnam; 7 Pre-Clinical Skills Training Center, Thai Binh University of Medicine and Pharmacy, Thai Binh City, Vietnam; 8 Surgery Department, Thai Nguyen University of Medicine and Pharmacy, Thai Nguyen City, Vietnam; 9 Harvard Medical School, Boston, Massachusetts, US; 10 Partnership for Health Advancement in Vietnam, Hanoi, Vietnam; 11 Harvard Medical School and Harvard T.H. Chan School of Public Health, Boston, Massachusetts, US; 12 Medicine Residency Program, Brigham and Women’s Hospital, Boston, Massachusetts, US

**Keywords:** primary health care, medical education, health workforce, collaboration, partnerships

## Abstract

Strong primary health care (PHC) systems require a robust PHC workforce. Traditionally, medical education takes place in academic medical centres that favour subspecialty care rather than PHC settings. This may undervalue primary care as a career and contribute to a shortage of PHC workers. However, designing undergraduate medical education curricula that incorporate early experiences in clinical care delivery at PHC sites remains a challenge, including in many low- and middle-income countries (LMICs).

This paper describes how a collaboration between Harvard Medical School and five medical schools in Vietnam, and in-country collaborations among the Vietnamese medical schools, facilitated curricular innovation and co-creation of coursework relevant to PHC through the development of a Practice of Medicine (POM) course. The collaboration implemented a technical assistance strategy consisting of in-person workshops, focused virtual consultations, on-site ‘office hours’, site visits and observations to each of the five medical universities, and immersion trips to support the creation and implementation of the POM course. A pilot program was started at a single site and then scaled nationally using local customisation, experience, and expertise utilising a train-the-trainers approach. As a result, five new POM courses have been developed by five Vietnamese institutions. Fifty Vietnamese faculty received training to lead the POM course development, and 228 community-based preceptors have been trained to teach students at PHC sites. A total of 52 new PHC and community-based clinical training sites have been added, and 3,615 students have completed or are currently going through a POM course. This experience can serve as a model for future academic collaborations to support the development of a robust PHC workforce for the 21st century.

## Introduction

A strong primary health care (PHC) system is essential to respond to shifting population health needs such as increases in non-communicable and chronic diseases, ageing populations, the health effects of climate change, and new emerging threats such as COVID-19 [1,2]. Health care systems with strong PHC are associated with better population health outcomes at a lower cost and with greater equity [3]. Strong PHC systems require a robust PHC workforce. The World Health Organization (WHO) estimates that there will be a shortage of 15 million health care workers by 2030, with PHC workers disproportionately affected [4]. This reality calls for medical education to focus on PHC [2,5,6,7,8].

Traditionally, the majority of medical education takes place in large academic medical centres (AMCs) with a focus on subspecialty and acute care rather than PHC [9]. The WHO proposes reforming undergraduate medical education (UME) curricula to increase PHC exposure [5]. Despite this call to action, many challenges remain, especially in low- and middle-income countries (LMICs) [10]. First, the scope and role of PHC varies by country, making best practices and successful models difficult to identify and transfer [11]. Second, PHC sites of care may be perceived as low status, quality, and prestige. This can translate to a ‘hidden curriculum’ that privileges training in AMCs over primary care or community-based sites and diminishes the value of primary care in the eyes of faculty and students [12,13]. Finally, in multiple countries, PHC is not considered a valued separate entity but rather a default pathway for those who do not pursue training in specialty care [14,15].

In LMICs which incorporate PHC competencies into UME, students value their experiences at PHC sites [9,16,17,18,19]. In countries with newly developing PHC curricula, a first step is introducing an elective in a PHC setting [20,21,22]. Beyond these elective experiences, there are few examples of UME curricula required for all students. In this paper we describe the development and implementation of a longitudinal course that horizontally and vertically integrates PHC experiences and concepts for all students. Our international team developed this course collaboratively as part of a larger national effort to reform UME in Viet Nam [23].

### Viet Nam context

Viet Nam, a lower middle-income country in Southeast Asia, has 29 medical universities with an average of 400–600 medical students matriculating per year at each school [23,24]. UME in Viet Nam is predominately a six-year program that follows the completion of secondary education [23,25]. Programmes are organised in a traditional format of the pre-clerkship years, consisting of basic science, followed by teaching of the clinical sciences and hospital-based rotations in the clerkship years [23,25]. In 1999, medical educators across Viet Nam collaborated to promote a community-centred approach to UME through the identification of learning objectives and outcomes expected of medical graduates [26,27,28,29]. In places where family medicine existed, universities created family medicine rotations [22]. The implementation of community-centred learning objectives predominately manifests as short periods where students participate in public health campaigns such as childhood immunization programmes and tuberculosis control [30]. These ‘stand-alone’ program components have limited integration into the general curriculum. Missing are direct experiences with patients in their community settings which allow students to integrate classroom learning into the real-world context.

### Reforming undergraduate medical education

In 2015, the Ministry of Health (MOH) committed to a national reform of UME grounded in competency-based medical education [30,31]. This reform refocuses medical education from the traditional approach of medical knowledge acquisition to training towards achievement of competencies based on population health needs. This reform began at five universities in Viet Nam. One reform objective is to enhance meaningful clinical experiences for students through early pre-clerkship opportunities. Several global UME reform efforts have accomplished early clinical exposure through having students learn communication skills, professionalism, and history taking through experiences with patients in PHC settings prior to starting their clerkships [32,33,34]. The deliberate design in which these skills are introduced in the pre-clerkship years in the PHC context has several advantages: (1) it provides meaningful authentic clinical experiences in PHC at an early stage in students’ careers, countering the hidden curriculum that diminishes the value of the PHC; (2) it allows students to integrate basic sciences with clinical experiences, deepening their learning; and (3) it allows students to enter their clerkship years with well-established clinical skills and professional values developed during the pre-clerkship years.

## Methods

Starting in 2015, the University of Medicine and Pharmacy at Ho Chi Minh City (UMP HCMC) initiated UME reform to include early clinical exposure through experiences with patients in PHC settings prior to starting their clerkships. Building from an existing relationship with Harvard Medical School (HMS), UMP HCMC faculty visited HMS to observe the pre-clerkship courses focused on early clinical exposure in PHC settings through integrated teaching of professionalism, history taking, physical exam, and communication skills [35]. In Viet Nam, these skills are often taught in skills labs and through observation of patient care in large groups in the hospital. Following the visit, HMS and UMP HCMC faculty collaborated to co-create a pre-clerkship course, the Practice of Medicine (POM). This course integrates the teaching of clinical skills and professionalism through student-patient encounters at PHC sites.

The POM curriculum was developed and pilot tested at UMP HCMC. Following extensive process evaluation and revision, the course was expanded to four additional medical universities, each adapting it to the local context. POM course development consisted of six components: (1) communication skills; (2) history taking skills; (3) physical exam skills; (4) professionalism; (5) affiliation with community sites through institutional agreements; and (6) faculty development. The following education and design principles guided all implementation efforts: vertical and horizontal integration, authentic contact with patients and practicing physicians, application of adult learning theory, evidence-based learning techniques, and commitment to continuous quality improvement (CQI). Please see Table 1 for additional details on curriculum integration.

**Table 1:** Technical assistance areas.


Communication skills	• Teaching communication skills • Writing learning objectives for communication skills • Assessment strategies to assess communication skills

History taking skills	• Teaching history taking • Writing learning objectives for history taking • Assessment strategies to assess history taking

Clinical skills	• Teaching discrete clinical skills • Writing learning objectives for clinical skills • Assessment strategies to assess clinical skills

Professionalism	• Introduction to professionalism • Teaching professionalism • Assessment strategies to assess professionalism

Affiliation with community/PHC sites	• Examples of models to expand clinical sites • Criteria and systems for expanding clinical sites • Distinguish roles and responsibilities of core and non-core faculty

Faculty development	• Integrating communication, clinical, and professionalism skills • Integrating basic sciences with clinical teaching • Developing teaching skills in community-based/PHC preceptors • Patient-based bedside teaching • Teaching clinical reasoning • Microteaching skills • Resident as teacher • Direct observation and feedback


Learning from the UMP HCMC pilot experience, we created general guidelines of requirements for clinical site selection (see Table 2), and each UMP established a UMP-specific checklist to select its own sites. Core faculty members and administrators met with key stakeholders at new sites to sign formal affiliation contracts between the PHC sites and the UMP. Incentives were offered to community-based clinicians who agreed to be preceptors, such as access to UMP educational resources, including continuous medical education (CME) courses and the UMP e-library system. In addition, community preceptors received formal affiliation status with the UMP. Since these incentives were financed by UMP operating budgets rather than funded with grants, they are sustainable beyond the funding period. UMP faculty trained community preceptors on precepting skills, and at the start of the program, they conducted on-site visits to PHC sites to observe and offer coaching support to community preceptors. Students were placed at these community sites as a part of mandatory coursework, often visiting the sites weekly or biweekly in accordance with the POM curriculum structure. Across all five UMPs, students and community preceptors completed course evaluations in the middle and at the end of the POM course.

**Table 2:** Suggested checklist for selecting community-based sites.


CRITERIA	YES	NO	NOTES

**Human resources**			

At least one physician on-site full time			

At least one physician who is licensed with at minimum two years of work experience and a medical teaching certificate			

At least one nurse who is licensed and has a minimum two years of work experience			

**Policies and protocols**			

There exist policies and protocols for:			

A. Infection control			

B. Needle stick injury			

C. Triage and transfer of sick patients to hospital-level care			

D. Reporting patient/clinic safety concerns			

**Operations and infrastructure**			

Sites are registered to accept government social health insurance.			

Sites have an active system of patient medical records (either paper based or electronic).			

The clinic can provide a dedicated space for student teaching.			

There is adequate AV equipment to support student teaching: projector, computer, whiteboard/blackboard, projection screen.			

The site has Wi-Fi.			

The clinic has organised an accessible system for frequently referenced medical texts and clinical support and protocols.			

The distance from the UMP and availability of public transport allow for easy routes to get from the UMP to the site.			


**Figure:**
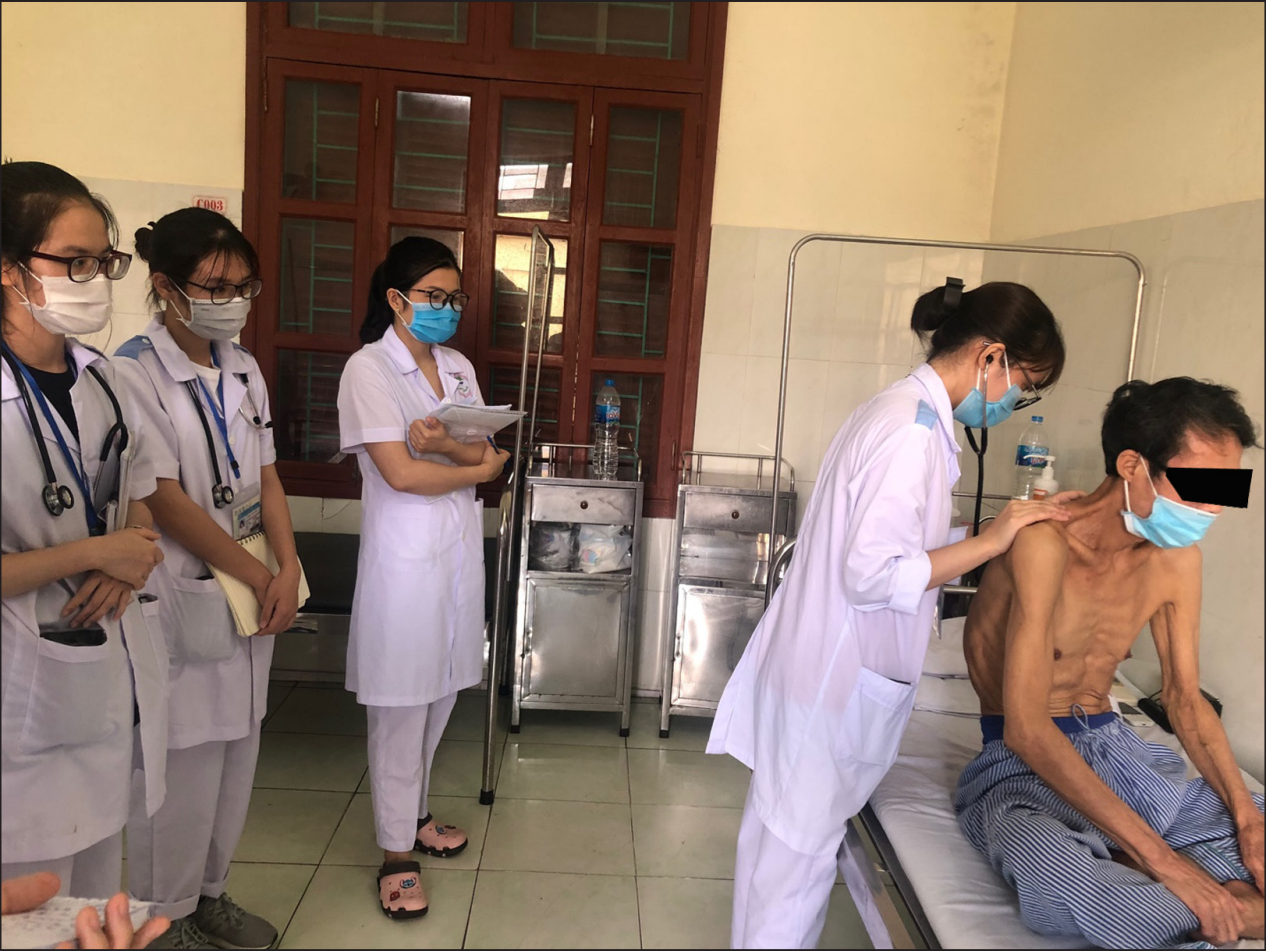
Students practicing the pulmonary examination on a patient a primary healthcare setting.

Technical knowledge transfer (herein ‘technical assistance’ (TA)) on the six components of the POM course enabled the adaptation and creation of individual POM courses at each UMP. TA consisted of in-person workshops, focused virtual consultations using Zoom videoconferencing, on-site ‘office hours’, site visits and observations to each of the five medical universities, and immersion trips for Vietnamese faculty to HMS to observe the HMS version of a POM course and to UMP HCMC to observe the local POM program for the remaining four universities. Funding for the universities to co-create and implement the POM course came from a World Bank loan; funding for HMS faculty participation came from a USAID grant. Next, we detail the process of our collaboration.

## Process of Collaboration

### Workshops

UMP and HMS faculty jointly determined workshop content through a collaborative process that began with a needs assessment and review of Ministry of Health–issued standard competencies for medical education. Workshops were delivered in-person with simultaneous translation. HMS faculty facilitated the initial workshops at UMP HCMC, and HMS and UMP HCMC faculty co-facilitated subsequent workshops for the four additional UMPs, establishing a train-the-trainer (TOT) model. Each participating UMP designated 10 ‘core faculty’ to participate in all TOT workshops. Workshops were delivered modelling the key principles of adult learning, and evidence-based teaching techniques were incorporated into the curriculum. Examples of these active-learning techniques include icebreakers, pair-share exercises, small group discussions, and role plays. We evaluated each workshop at the end of each workshop day to rapidly respond to participants’ input and feedback. During these workshops, we also encouraged networking across the UMPs to increase collaboration and shared learning and to build a national community of medical educators.

### Office hours and site visits

In-person ‘office hours’ followed each workshop to allow individual universities to present work-in-progress and site-specific implementation challenges. Office hours were accompanied by site visits, during which HMS faculty visited new PHC sites, participated in faculty development, and directly observed POM teaching practices. Each UMP then established interim program goals to be monitored virtually until the next workshop.

### Virtual sessions

Vietnamese and HMS faculty provided virtual TA sessions via the Zoom videoconferencing platform. These took place at regular intervals between in-person workshops and site visits. These sessions allowed for ongoing feedback on learning objectives, course guides, plans for faculty development, and plans for implementation.

### Immersion trips

Immersion trips to HMS from UMP HCMC and among the four universities to UMP HCMC provided opportunities for core faculty to observe various versions of the POM course and interact with faculty and students. These trips also allowed core faculty to see examples of successful implementation of POM at PHC sites and provided opportunities for Vietnamese faculty to share their expertise and best practices.

The combination of group and individual TA allowed for all UMPs to share core educational content (i.e., six components and the four core educational principles) as well as address specific contextual challenges at each UMP, such as infrastructure and availability of PHC sites. For example, some UMPs extended the pre-clinical POM course from two years to three years, while others with more limited faculty and sites teach selected content in skills labs. All UMPs expanded teaching of POM content throughout the clerkship years, including continuing student engagement with PHC sites.

## Results

Since the initiation of this work, 5 new POM courses have been developed by 5 Vietnamese institutions, 50 Vietnamese faculty have been trained to lead the POM course development, and 228 community-based preceptors have been trained to teach students at their PHC sites. A total of 52 new PHC and community-based clinical training sites have been added, and 3,615 students have completed or are currently enrolled in a POM course. All 5 new POM courses have implemented a CQI approach for ongoing reform, which includes a standardised process to solicit student feedback and incorporate it into future iterations of the course. Finally, through this intensive, collaborative approach, we created a learning community among the core faculty at and across the UMPs.

## Discussion

During the implementation of the POM course, each UMP faced unique challenges in expanding clinical sites to PHC and the community. A key challenge has been accommodating the large number of pre-clerkship students and providing them with adequate hands-on clinical experiences. Solutions included a hybrid approach, combining skills labs, role plays, and video vignettes in combination with direct patient experiences; cohorting students; offering POM sessions to a smaller number of students; and extending POM curriculum over three years to ensure adequate exposure for all students.

Our greatest challenge was and continues to be the variable quality of PHC facilities and inconsistencies in the complexity and volume of patients, resulting in inconsistent and unpredictable clinical learning opportunities for students [30,36]. In addition, POM course directors were hesitant to send students to rotate through sites where they did not know the skill and knowledge levels of the preceptors. To address this, participating UMPs established faculty development training for preceptors focusing on the key domains of POM. They refined POM learning objectives and clarified which were best learned in PHC sites. Faculty development efforts and capacity have expanded at each UMP and provide for ongoing improvement in teaching skills for all clinical faculty, including preceptors in PHC settings. This led to PHC preceptors’ development in the four core domains of POM: clinical skills, history taking, professionalism, and communication skills. Additionally, engagement in teaching may enhance the knowledge and skills of a practicing clinician; therefore, a potential benefit of integrating PHC sites into UME is that the PHC system may be strengthened, although the immediate direct strengthening of the PHC system is not a goal of our project.

Despite the challenges, the incorporation of community sites into the UME curriculum created two opportunities. First, health systems–based education initiatives correspond to the third generation of health professional education reforms and are a necessary area of competence for medical students [9,37]. The POM course completes the students’ health care delivery experiences, starting with care delivery at the community level and in the later years at tertiary AMCs. Second, in the Viet Nam context, increasing the number of PHC sites decreases the number of students rotating through AMCs, increasing opportunities for direct experiences with patients, and decreases the student-to-faculty ratio through the recruitment of PHC preceptors.

The creation of a core group of faculty at each UMP and between the UMPs establishes learning communities that share experiences and lessons to continue to develop the POM course. Embedding the newly created POM course into a larger national curriculum reform effort, and establishing CQI processes, ensures not only the sustainability but also further improvements and evolution of the POM course at the UMPs.

### Limitations

Given how new and recent our program is across the five UMPs (i.e., the first cohort of students who have completed the POM course have not yet graduated), we do not have data on students’ intentions to pursue a career in primary health care, graduation rates, or other indicators that may measure the impact of this course on the overall PHC workforce in Viet Nam. In addition, as attention was concentrated on building the program and implementing it across the five UMPs, there was limited focus on a deep analysis of the effects of the program on students and the systems they work in. Further research and analysis on these areas will be critical as this program continues in each of the five UMPs and spreads to other medical schools in Viet Nam. Finally, while there may be similarities among the medical education structures in various LMIC countries, the extent to which this model is transferable is not yet known.

## Conclusion

Our work is a part of a comprehensive reform of UME in Viet Nam that incorporates PHC experiences on a national scale. Our experience in Viet Nam demonstrates that with local leadership and ownership, coupled with dedicated TA from experienced institutions, integration of experiences at PHC sites is possible in LMICs, not just as pilots but as major components of the UME curriculum. Our collaboration was possible because it was built on existing long-term relationships between HMS and the UMPs and the Viet Nam Ministry of Health which span over 15 years and have resulted in many shared successes and impacts on the Vietnamese health care sector, most notably in HIV/AIDS care and treatment. We recognise that there are no shortcuts to building trust, communication, and shared purpose. Our work benefitted from this intangible but critical foundation. Our model of collaboration can be further locally scaled to the remaining medical universities in Viet Nam and possibly other LMICs. We recommend that the impact on the PHC workforce in Viet Nam be tracked as this program continues to be implemented. Our hope is that with a solid foundation of PHC education that is universal to all medical students across the five UMPs, there will be a positive shift in the value placed on PHC and further investments in PHC sites and the PHC workforce.

## Authour Information

All authors had access to the data and a role in writing the manuscript.

David B. Duong and Tuan A. Nguyen are co-first authors.
